# Is monocyte- and macrophage-derived tissue transglutaminase involved in inflammatory processes?

**DOI:** 10.1007/s00726-016-2334-9

**Published:** 2016-09-22

**Authors:** Navina L. Chrobok, Claudia Sestito, Micha M. M. Wilhelmus, Benjamin Drukarch, Anne-Marie van Dam

**Affiliations:** Amsterdam Neuroscience, Department of Anatomy and Neurosciences, VU University Medical Center, De Boelelaan 1118, 1081 HV Amsterdam, The Netherlands

**Keywords:** Inflammation, Adhesion, Differentiation, Efferocytosis, Multiple sclerosis

## Abstract

Monocytes and macrophages are key players in inflammatory processes following an infection or tissue damage. Monocytes adhere and extravasate into the inflamed tissue, differentiate into macrophages, and produce inflammatory mediators to combat the pathogens. In addition, they take up dead cells and debris and, therefore, take part in the resolution of inflammation. The multifunctional enzyme tissue Transglutaminase (TG2, tTG) is known to participate in most of those monocyte- and macrophage-mediated processes. Moreover, TG2 expression and activity can be regulated by inflammatory mediators. In the present review, we selectively elaborate on the expression, regulation, and contribution of TG2 derived from monocytes and macrophages to inflammatory processes mediated by those cells. In addition, we discuss the role of TG2 in certain pathological conditions, in which inflammation and monocytes and/or macrophages are prominently present, including atherosclerosis, sepsis, and multiple sclerosis. Based on the studies and considerations reported in this review, we conclude that monocyte- and macrophage-derived TG2 is clearly involved in various processes contributing to inflammation. However, TG2’s potential as a therapeutic target to counteract the possible detrimental effects or stimulate the potential beneficial effects on monocyte and macrophage responses during inflammation should be carefully considered. Alternatively, as TG2-related parameters can be used as a marker of disease, e.g., in celiac disease, or of disease-stage, e.g., in cancer, we put forward that this could be subject of research for monocyte- or macrophage-derived TG2 in inflammatory diseases.

## Monocytes and macrophages in inflammatory processes

### What is inflammation?

When harmful stimuli, e.g., infectious agents, are encountered by the body, inflammation is the initial protective response (Murphy et al. 2008). The classical signs of acute inflammation are heat, pain, redness, swelling and loss of function. Inflammation is a generic response, and, therefore, is considered as a mechanism of innate immunity. Pathologically, the inflammatory response is regulated by the release of chemotactic and vasoactive mediators and characterized by the movement of leukocytes from the blood into the affected tissue. Exposure to harmful stimuli results within minutes up to hours in an acute inflammatory response with the aim of counteracting or restricting the damage induced on the body. If the resolution of inflammation is disturbed, or persistent exposure to harmful stimuli is present, the acute inflammation might result in chronic inflammation, which is defined by concurrent damaging and healing processes.

The secretion of inflammatory mediators from the inflamed tissue leads to vasodilation and increased permeability of the blood vessels, facilitating extravasation of immune cells into the inflamed tissue. This acute inflammatory response is mediated by the early influx of granulocytes, mainly neutrophils, followed by monocytes that subsequently differentiate into inflammatory macrophages. A classical example of acute inflammation is septic shock (Reinhart et al. 2012). Chronic inflammation is a pathological state characterized by the persistent presence of lymphocytes, monocyte, and macrophages, and is a defining characteristic of various diseases, including atherosclerosis (Schwartz et al. 1992), rheumatoid arthritis (Roberts et al. 2015), and multiple sclerosis (MS) (Sospedra and Martin 2005).

### The roles of monocytes and macrophages during inflammation

During the inflammatory process, monocytes and macrophages are attracted to the site of damage, where they participate in a variety of processes that result in the stimulation or resolution of inflammation. The most important processes in which they take part will be discussed in the following paragraphs.

#### Adhesion and extravasation

Two main processes in the early phases of inflammation exerted by monocytes are adhesion and extravasation. Monocytes are drawn to the site of inflammation by the chemotactic gradient of inflammatory factors released at the inflamed site. This facilitates their adherence to the activated vascular endothelium, followed by extravasation and migration through the tissue (Gerhardt and Ley 2015). In more detail, monocytes in the bloodstream first roll on the activated and/or inflamed endothelial lumen, before they strengthen their adherence and crawl along the lumen to find an extravasation site into the inflamed tissue (Ley et al. 2007; Gerhardt and Ley 2015). Monocyte adhesion is dependent on a plethora of cell surface molecules (Gerhardt and Ley 2015). A well-known group of molecules that is involved in these processes is the family of integrins. Integrin heterodimers expressed on monocytes are involved in the arrest and initial adhesion of these cells to the vascular endothelium by binding to endothelial cell adhesion molecules (Laudanna et al. 2002; Ley et al. 2007). This process facilitates the extravasation of these cells from the bloodstream into the tissue.

#### Monocyte differentiation into macrophages

Blood monocytes recruited to inflammatory sites differentiate into inflammatory macrophages with distinct functions and act as key players in the innate immune response (Gordon and Taylor 2005; Yang et al. 2014).

Macrophages can adopt diverse activation states in response to inflammatory stimuli. The two extreme sides of the polarization spectrum are nowadays referred to as classically activated (M1) macrophages and alternatively activated (M2) macrophages (Mosser and Edwards 2008). In general, the M1 phenotype is regarded to represent a pro-inflammatory activation state, whereas the M2 phenotype is indicative of cells displaying anti-inflammatory properties (Gordon 2003). This classification of polarized phenotypes is based on in vitro experiments. Unfortunately, it is often not transferrable to the actual in vivo situation. Although both phenotypes can also be identified in vivo, the majority of these cells exhibit intermediate polarization states (Martinez et al. 2009). This phenomenon is also reflected during inflammation when M1, M2 and intermediate macrophage phenotypes are present simultaneously and each exerts their specific functions. Nonetheless, the M1 macrophage phenotype is predominantly required in the early stages of inflammation when it releases inflammatory mediators that further stimulate inflammation and attract leukocytes (Yang et al. 2014). Later, in the inflammatory response, the M2 macrophage phenotype exhibits phagocytic and efferocytic functions, supports tissue repair, and thereby helps to resolve the inflammatory process (Martinez et al. 2009).

#### Phagocytosis and efferocytosis

Resolving of the inflammatory process occurs during what is known as the resolution phase. During this phase of inflammation, M2 macrophages clear pathogens (in the case of infections), necrotic debris, and apoptotic cells via phagocytic uptake. These actions reduce the source of inflammation and are accompanied by the secretion of anti-inflammatory mediators, e.g., transforming growth factor beta1 (TGF-β1) and platelet-activating factor (PAF), and overall results in the resolution of the inflammatory response. The clearance of apoptotic cells by efferocytosis, a specific form of phagocytosis, is crucial by preventing the secondary necrosis and release of inflammatory mediators leading to continued tissue damage and inflammation (Savill 1997; Fadok et al. 1998; Savill et al. 2002). Furthermore, efferocytic macrophages actively repress inflammation by increased expression of anti-inflammatory mediators and reduced expression of pro-inflammatory mediators, e.g., interleukin 1 beta (IL-1β) and tumour necrosis factor alpha (TNF-α) (Fadok et al. 1998; Lawrence et al. 2002).

## Tissue transglutaminase

Tissue transglutaminase (tTG, TG2) is a multifunctional enzyme that was first described in the late 1950s (Sarkar et al. 1957; Mycek et al. 1959). Upon discovery in guinea pig liver, it was demonstrated to be able to catalyse protein–protein crosslinking. Later on, TG2 turned out to be a multifactorial enzyme associated with important functions in both physiological and pathological conditions.

The family of transglutaminases, including TG2, consists of structurally and functionally related enzymes and is best known for their calcium dependent transamidation function, which is present in eight of the family members (Lorand and Graham 2003). This transamidation function catalyses the crosslinking of glutamine to lysine residues within or between proteins, resulting in a stable isopeptide bond (Lorand and Graham 2003). This means that the cross-linked substrates are not only more protease resistant, but also they are also known to result in altered biological functioning, metabolism, and/or immunogenicity of the substrates (Lorand and Graham 2003). Moreover, amine incorporation into substrates and deamidation are catalysed by the same enzymatic domain (Schuppan and Hahn 2002; Lorand and Graham 2003).

Alike some other members of the family, TG2 has various additional enzymatic functions, i.e., disulfide isomerase function (Hasegawa et al. 2003), G protein (GTPase) function during which TG2 is better known as Ghα (Achyuthan and Greenberg 1987; Nakaoka et al. 1994) and protein kinase activity (Mishra and Murphy 2004). However, these functions are not the focus of this review, as their participation in the processes and functions of monocytes and macrophages discussed here is mostly unknown.

The molecular structure of TG2 consists of four domains that are involved in distinct functions: the N terminal β-sandwich (binds fibronectin and integrins), the catalytic core (transamidating activity), and two C terminal β-barrels of which the latter includes a phospholipase C-binding sequence (Fesus and Piacentini 2002).

As various as its alleged functions are the reported subcellular locations of TG2. Although predominantly present in the cytoplasm, TG2 has also been found in the nucleus, mitochondria, endoplasmatic reticulum, and on the cell surface (Lesort et al. 1998; Balajthy et al. 2006; Hodrea et al. 2010; Wilhelmus et al. 2011; Nurminskaya and Belkin 2012). Furthermore, TG2 can be secreted from the cell into the extracellular matrix (ECM) (Aeschlimann and Thomazy 2000; Lorand and Graham 2003; Park et al. 2010).

TG2 is constitutively expressed in many tissues and cell types, amongst others in monocytes and macrophages, and is upregulated in a cell-type-dependent manner by several physiological and pathological stimuli. Increased TG2 expression and transamidation activity, also known as protein cross-linking activity, are often observed under inflammatory conditions in which cytokines and growth factors, released by injured cells, regulate TG2 expression and activity (Iismaa et al. 2009).

Another well-characterized member of the transglutaminase family that is likewise present intracellularly and on the surface of monocytes is FXIII. The secreted form FXIIIa is best known for its function in the blood coagulation cascade, where it cross-links fibrin and forms a lysis resistant blood clot that facilitates wound closure (Lorand et al. 1993). The presence and possible involvement of FXIIIa in certain processes has to be taken into account when studying the role of TG2 in circulating monocytes or monocyte-derived macrophages.

TG2, with its highly diverse functions and locations, is in an ideal position to contribute to a variety of cellular events, such as cell adhesion and migration (Akimov and Belkin 2001b), cell differentiation (Leu et al. 1982), and efferocytosis (Fesus et al. 1981).

## TG2 function in monocytes and inflammatory macrophages

### TG2 in cell adhesion and extravasation

The participation of TG2 in cell adhesion and extravasation, processes crucial for monocytes to reach the site of inflammation, was first shown for fibroblasts and later for several other cell types, including monocytes (Gentile et al. 1992; Akimov and Belkin 2001b). The protein fibronectin is a major constituent of the ECM and serves as a main substrate for adhesion and migration of cells (Pankov and Yamada 2002). Integrins on the cell surface bridge the intracellular cytoskeleton with fibronectin in the ECM and thus facilitate adhesion of cells onto the ECM. (Pankov and Yamada 2002). The contribution of TG2 to adhesion was first described as the interaction of TG2 with fibronectin (Turner and Lorand 1989). Accordingly, the fibronectin-binding domain in the TG2 structure was defined, and as a result, fibronectin is the best studied substrate for TG2 action in adhesion and migration (Akimov et al. 2000).

The reduction of TG2 production by TG2 knock-down or inhibition of TG2-fibronectin binding by function-blocking antibodies strongly reduced the cellular adhesion and migration capacities of macrophages (Akimov and Belkin 2001b). Special emphasis was set on investigating the participation of FXIIIa in these processes, as it is present in monocytes. However, no contribution of FXIIIa to adhesion and/or migration processes could be shown, so that the above-mentioned effects are probably due solely to TG2 (Muszbek et al. 1996; Falasca et al. 2005; Toth et al. 2009). Moreover, when monocytes differentiated into macrophages upon adhesion, the cellular expression of TG2 increased, whereas the FXIIIa expression decreased, making it unlikely that FXIIIa is of relevance in cell adhesion processes of monocytes and macrophages (Seiving et al. 1991; Akimov and Belkin 2001b).

Cell adhesion onto other cells or the ECM includes integrin binding. TG2 on the cell surface of macrophages is complexed with integrins, i.e., β1/β3/β5-integrins (Akimov and Belkin 2001b). Moreover, increased TG2 expression in macrophages results in a simultaneous increase in β-integrin expression and presence on the cell surface. These TG2-integrin complexes function as a bridge between integrins and fibronectin on cell surfaces and in the ECM, facilitating cell adhesion (Akimov et al. 2000). In addition, the localization of cell surface TG2-integrin complexes in specialized adhesive structures in macrophages called podosomes, point towards a strong link between TG2-integrin complexes and adhesion and extravasation of the cell (Marchisio et al. 1987; DeFife et al. 1999). Interestingly, the interaction between TG2 and β-integrins does not require transamidation activity to increase adhesion, spreading, and cell motility (Gaudry et al. 1999; Akimov et al. 2000; Akimov and Belkin 2001a; Balklava et al. 2002; Lorand and Graham 2003; Huelsz-Prince et al. 2013). Thus, although the transamidation activity of TG2 is unlikely to play a role in the adhesion and migration processes, the formation of the above-mentioned specific protein complexes appears crucial. Therefore, more research is needed to further define the precise nature of the interaction of TG2 and other ECM proteins to determine the mechanisms of action in cell adhesion and migration.

In addition to its involvement in cell binding to ECM proteins, TG2 is also an important player in assembly, remodelling, and stabilization of the ECM via crosslinking of several ECM substrates, in particular fibronectin, vitronectin, von Willebrand factor, proteoglycans, and collagens (Aeschlimann and Thomazy 2000; Griffin et al. 2002). ECM stabilization by TG2-mediated transamidation increases the rigidity of the ECM. This rigidity can selectively trigger focal adhesion formation and may hence result in increased cell adhesion to the ECM (Sheetz et al. 1998; Giannone and Sheetz 2006). Moreover, the crosslinking of ECM proteins increased the clustering of binding sites for cells to adhere to the ECM (Miyamoto et al. 1995; Lo et al. 2000).

The above described contribution of TG2 to adhesion and migration appears to be mediated by cell surface and/or extracellular TG2. Migration and especially diapedesis of cells through the blood vessel endothelium require a high motility of the actin cytoskeleton to be able to change the shape of the cell in an efficient and fast manner. This reorganization of the cytoskeletal structure is mediated by focal adhesion kinase (FAK) and Rho-associated protein kinase (ROCK) (Honing et al. 2004; Schnoor 2015). Both of these are stimulated by TG2-induced clustering of cell-surface integrins, which lead to higher initiation of cell adhesion and cytoskeletal flexibility (Janiak et al. 2006). In line with this, it has been reported that RhoA activity, an upstream stimulator of ROCK, was reduced after the inhibition of TG2 transamidating activity in rat macrophages and this coincided with reduced F-actin cytoskeletal rearrangement (van Strien et al. 2015).

Interference of the specific protein-complex formation of TG2 with integrins and fibronectin in the process of adhesion, extravasation and migration could potentially serve as a pharmacological target to modulate these cellular processes. Recently, such a manipulation has been described to prevent cancer metastasis by interfering with cell adhesion onto fibronectin and likewise migration (Khanna et al. 2011; Yakubov et al. 2014).

### TG2 in the differentiation of monocytes into macrophages

To exploit their functions in the inflamed tissue, circulating monocytes need to adhere, migrate, and ultimately differentiate into mature tissue macrophages. The molecular mechanism by which monocytes undergo this morphological and functional differentiation during inflammation is not fully characterized yet. However, it has been proposed that TG2 plays a role in this process. This proposal is based on the in vitro observations that TG2 expression and cross-linking activity are low in freshly isolated monocytes, but exponentially increased during the maturation process of monocytes, induced either chemically or by adherence to the cell-culture dish (Murtaugh et al. 1984; Mehta and Lopez-Berestein 1986; Metha et al. 1987). Moreover, in this context, it may be of importance that the upregulation of TG2 expression in monocytes represents a constitutive process which takes place under physiological conditions, e.g., after monocyte adhesion onto endothelial cells (Thomas-Ecker et al. 2007). During adhesion onto naïve endothelial cells, monocytes upregulate genes required for the transendothelial migration and initiate the differentiation program into phagocytes. This suggests that TG2, as one of the highly expressed genes, can play a role in the maturation process (Thomas-Ecker et al. 2007). In addition, although the classification of tissue macrophages is still under debate, TG2 upregulation seems to occur predominantly in alternatively activated M2 macrophages (Martinez et al. 2013; Eligini et al. 2015). In fact, this observation recently led to the suggestion of TG2 as a specific M2 macrophage marker (Martinez et al. 2013).

### TG2 in efferocytosis

Clearance of apoptotic cells by efferocytosis is crucial for the resolution of inflammation by preventing the secondary necrosis and release of inflammatory mediators (Savill 1997; Fadok et al. 1998). The involvement of TG2 in efferocytosis was already established in 1981, and TG2 was suggested to play a role in the efferocytic capacity of macrophages (Fesus et al. 1981; Leu et al. 1982; Murtaugh et al. 1984; Mehta and Lopez-Berestein 1986; Nadella et al. 2015), which was generally absent in undifferentiated monocytes with little TG2 expression (Mehta and Lopez-Berestein 1986). Therefore, TG2 in macrophages is considered as an important mediator of efferocytosis that limits inflammation by the removal of apoptotic cells and additionally decreases pro-inflammatory cytokine secretion, both of which are impaired in the absence of TG2 or when TG2 activity is inhibited (Szondy et al. 2003; Falasca et al. 2005; Rose et al. 2006; Sarang et al. 2011; Toth et al. 2009; Nadella et al. 2015; Eligini et al. 2016). Furthermore, defects in efferocytosis are also linked to autoimmunity (Shao and Cohen 2011). Indeed, TG2 knockout (TG2^−/−^) mice demonstrate signs of autoimmunity due to insufficient efferocytosis with increasing age, supporting again TG2 as a mediator of efferocytosis (Szondy et al. 2003). Of interest is that macrophages in TG2^−/−^ mice show specifically reduced efferocytosis of apoptotic leukocytes but not of phagocytosis per se, as shown for bacteria, yeast, opsonized non-apoptotic thymocytes, or monosodium urate crystals (Szondy et al. 2003; Rose et al. 2006; Toth et al. 2009). This indicates that TG2 present in macrophages does not participate in phagocytosis of particles or debris.

Efferocytosis is mediated by the formation of a phagocytic cup that tethers apoptotic cells to macrophages and facilitates their engulfment (Toth et al. 2009). It has been reported that a lack of TG2 in macrophages diminishes efferocytosis by reduced engulfment and does not abridge tethering of apoptotic cells (Falasca et al. 2005). However, other data indicated that TG2 can indirectly be involved in the tethering of apoptotic cells (Toth et al. 2009). β3-integrin promotes the tethering of apoptotic cells to macrophages (Savill et al. 2002), and TG2 induces *β*3-integrin clustering on the cell surface in a phagocytic cup (Toth et al. 2009). Therefore, a lack of TG2 results in a less efficient formation of phagocytic portals which leads to reduced tethering and engulfment of apoptotic cells (Toth et al. 2009).

TG2-mediating efferocytosis has so far been linked to extracellular or cell surface localization of the enzyme. This is supported by corrected in vitro efferocytosis in TG2^−/−^ macrophages after adding exogenous recombinant TG2, either wild type or catalytically inactive enzyme (Rose et al. 2006). Likewise, the functions of TG2 in efferocytosis are independent of GTPase activity (Rose et al. 2006).

Taken together, although the participation of TG2 in efferocytosis via interaction with integrins has clearly been established, the precise nature of the mechanisms involved is still a topic of research.

## TG2 and inflammation

As described, TG2 is contributing to various processes that occur in monocytes and/or macrophages during inflammation, including cell adhesion, extravasation, and efferocytosis. The remaining question is whether TG2 produced by macrophages is regulated by inflammation or inflammation-related mediators, e.g., cytokines.

### Inflammatory mediators

During inflammation, various inflammatory mediators, including cytokines, are produced to modulate the cellular responses involved. To experimentally induce an inflammatory status, lipopolysaccharide (LPS), the major component of the outer membrane of Gram-negative bacteria, is often used. It has been shown that LPS increases TG2 mRNA expression and activity in macrophages (Hayakawa et al. 2016) and BV-2 microglial cells [the resident macrophages of the central nervous system (CNS)] (Park et al. 2004; Kawabe et al. 2015). The induction of TG2 production was closely associated with enhanced phagocytic properties of microglia and nitric oxide production (Kawabe et al. 2015), mediated via LPS-induced activation of the NFκB pathway (Park et al. 2004). The promotor region of TG2 contains certain cytokine and NFκB responsive elements (Lu et al. 1995; Mirza et al. 1997; Ritter and Davies 1998), thus when inflammation occurs and the NFκB pathway is activated, this may up-regulate TG2 expression. Once induced, TG2 can in turn contribute to further NFκB activation by crosslinking the inhibitory molecule IKBα, leading to a sustained expression of various target genes involved in inflammation, such as inducible nitric oxide synthase and TNF-α (Lee et al. 2004). This activation loop has also been demonstrated in a mouse macrophage cell line in which LPS treatment induced the production and activation of TG2, NFκB, and metastatic tumour antigen 1 (MTA1, a master chromatin modifier). The latter interacts with NFκB to induce TG2 gene expression (Ghanta et al. 2011). Besides LPS, the pro-inflammatory cytokine interferon gamma (IFN-γ) is an inducer of TG2 production in monocytes (Mehta et al. 1985, 1987). Although other pro-inflammatory mediators, such as TNF-α and IL-1, are able to induce TG2 expression in different cell types, including liver cells, chondrocytes, and astrocytes (Kuncio et al. 1998; Johnson et al. 2001; van Strien et al. 2011), no literature is available regarding the regulation of TG2 expression by these mediators in monocytes or macrophages.

In contrast to its onset, the resolution of inflammation is mediated by the secretion of anti-inflammatory cytokines, such as interleukin-4 (IL-4) and TGF-β, which can induce tissue repair. Intriguingly, in apparent contrast to the above-mentioned pro-inflammatory regulation of TG2, the regulation of monocyte and macrophage-derived TG2 by anti-inflammatory mediators has been recently proposed as well. Indeed, IL-4 treated primary human macrophages up-regulate TG2 gene expression in vitro (Gratchev et al. 2005; Yamaguchi et al. 2016). In addition, using a proteomic approach, TG2 was identified as a novel M2 marker induced in human and mouse macrophages (Martinez et al. 2013). Moreover, the induction of an M2 phenotype in rat macrophages coincides with an upregulation of TG2 expression (Hayakawa et al. 2016). In line with these findings, it has been reported that *Glycine tomentella Hayata*, a herbal medicine with anti-inflammatory properties, upregulates TG2 expression in RAW264.7 mouse macrophages and enhances the clearance of apoptotic cells (Yen et al. 2010). In addition to IL-4, TGF-β exerts pro-resolution activity during an inflammatory response. Although TGF-β-induced TG2 expression is very well established in various cell types, such as fibroblasts or human trabecular meshwork cells (Jung et al. 2007; Tovar-Vidales et al. 2011), thus far no studies report regulation of TG2 expression by TGF-β in monocytes and macrophages. Alternatively, TG2 has been described to mediate TGF-β production in these cells, clearly indicating a link between the two (Hsu et al. 2007).

IL-6 has been shown to positively regulate TG2 expression in macrophages. The treatment of human THP-1-derived macrophages with IL-6 induced almost a twofold upregulation of TG2 mRNA expression paralleled by an increase in the production of anti-inflammatory cytokines and efferocytosis rate (Frisdal et al. 2011). All together, these observations indicate that TG2 production and/or activity can be regulated in macrophages during inflammatory processes, irrespective of the pro- or anti-inflammatory nature of the mediators involved.

## TG2 in inflammatory diseases

In various human disorders, TG2 has been shown to underlie or contribute to disease progression. This ranges from coeliac disease (Rauhavirta et al. 2016) and cancer (Agnihotri and Mehta 2016) to neurodegenerative diseases, e.g. Alzheimer’s disease (Wilhelmus et al. 2014). However, monocytes and macrophages are not generally considered as central players in their pathogenesis. So far, we discussed TG2 expression in monocytes and macrophages that mediate several cellular processes (e.g. adhesion and efferocytosis) involved in the inflammatory response. We will now discuss the contribution of monocyte and macrophage-derived TG2 to the inflammatory processes underlying atherosclerosis, sepsis, and MS.

### Atherosclerosis

Atherosclerosis is a vascular disorder in which fatty substances and cholesterol form a deposit (plaque) on the inside of the arterial walls. Both inflammatory mediators and macrophages play a prominent role in the development of atherosclerotic plaques, and thus, inflammation-induced TG2 can possibly play a role in the pathogenesis of atherosclerosis (Moore et al. 2013). It has been shown that macrophage-derived TG2 functions as an endogenous apoptotic cell clearance and anti-inflammatory factor that limits the expansion of atherosclerotic plaques by inducing TGF-β activation in macrophages (Boisvert et al. 2006). In addition, TG2 meditates ECM protein crosslinking resulting in stable plaque formation (Van Herck et al. 2010). Moreover, TG2 deficiency fundamentally impairs the activation of TGF-β and the capacity of macrophages to ingest apoptotic cells (Boisvert et al. 2006). It has also been suggested that TG2 can play a role in the stabilization of the structure of the dying cells and, therefore, in the prevention of the leakage of harmful cell content (Van Herck et al. 2010). The anti-inflammatory function of TG2 in atherosclerotic plaques seems to be modulated by different mediators, including estrogen receptor alpha (ER-α), which has been identified as a direct regulator of TG2 expression. Furthermore, an estrogen response element (ERE) was identified in the promoter region of TG2 (Ribas et al. 2011) supporting the regulation of TG2 by ER agonists.

Conversely to this protective role of macrophage-derived TG2 in limiting the atherosclerotic plaque expansion, it has been shown that the systemic inhibition of both TG2 and FXIIIa resulted in 41 % less macrophages infiltrating the media of the vessel. Since it is known that the development of atherosclerosis involves the production of reactive oxygen species (ROS) mainly by macrophages, the observed reduction in macrophage number could reflect a reduction in the production of ROS. Those findings propose a contributing role for TG2 in the early development of plaque formation (Matlung et al. 2010).

Taken together, the role of macrophage-derived TG2 in atherosclerosis pathogenesis possibly depends on the phase of the disease, as it can support the early development of lesion formation and act as a protective factor in the stabilization of atherosclerotic plaques at a later stages.

### Septic shock

Sepsis is a clinical syndrome caused by an exaggerated inflammatory response to infection (O’Brien et al. 2007). During sepsis, the activation of the NFκB pathway has been shown to play a central role (Liu and Malik 2006). The mutual regulation between TG2 and NFκB pathways during an inflammatory response is indicative of TG2 being a potential contributor to the pathogenesis of sepsis. In wild-type mice in which septic shock was induced by LPS treatment, the induction of macrophage-derived TG2 leads to a loop of continuous activation of the NFκB pathway, which in turn contributes to a constitutive production of pro-inflammatory cytokines ultimately resulting in organ failure. Conversely, due to the transient activation of NFκB, TG2^−/−^ mice are partially resistant to experimental sepsis elicited by LPS treatment, resulting in a reduction in liver injury (Falasca et al. 2008). Moreover, the observed downregulation of Toll-like receptor 4 (TLR4, LPS receptor) in dendritic cells derived from TG2^−/−^ mice seems to add on to the resistance to LPS-induced septic shock (Matic et al. 2010). Interestingly, another study in which TNF-α/Actinomycin-D was used to induce septic shock, resulting in apoptosis of liver cells, showed opposite results. TG2^−/−^ mice seemed to be more prone to induced liver injury compared to wild-type mice (Delhase et al. 2012; Yoo et al. 2013). This is probably due to enhanced caspase-3 expression and subsequent elevated apoptosis of liver cells in the absence of TG2.

Taken together, these findings point towards al role for TG2 in the pathogenesis of septic shock, although whether it is detrimental or beneficial appears to be dependent on the type of stimulus inducing septic shock and the cells/tissues affected.

### Multiple sclerosis

The involvement of monocyte- and macrophage-derived TG2 in the pathogenesis of MS is an interesting and unexplored new field. MS is a disease of the CNS, clinically characterized mostly by sensory, motor, and cognitive impairment (Noseworthy et al. 2000). Pathologically, leukocyte infiltration, demyelination, and ultimately axonal loss can be observed (Lassmann 2011). Although generally considered as a chronic inflammatory disease, the most common form of MS is characterized by episodes of acute inflammation, which leads to the destruction of the blood brain barrier resulting in influx of immune cells into the CNS, followed by the remission of inflammation (Noseworthy et al. 2000). Our group was the first to observe the appearance of immunoreactive TG2 in infiltrated macrophages in white matter lesions of MS patients (van Strien et al. 2015). In addition, TG2 appeared in the CNS of marmosets suffering from experimental autoimmune encephalomyelitis (EAE), a non-human primate model for MS (Espitia Pinzon et al. 2014). In active white matter lesions in these animals, TG2 immunoreactivity was observed in round-shaped cells localized in proximity to the blood vessel. These cells co-localized with Iba1, a marker for macrophages and microglia. Furthermore, due to its co-localization with β1-integrin and the close association with extracellular fibronectin, we put forward that TG2 may play a prominent role in the adhesion and migration of infiltrating monocytes and macrophages during EAE. Noteworthy is the fact that TG2 seems to be differentially expressed at various stages of lesion activity. In particular, the number of TG2 positive cells reduced when the lesions lose activity. Conversely to the white matter lesions, in cortical grey matter lesions, fibronectin expression is absent and TG2 seems to be predominantly expressed by resident microglia.

The role of TG2 in the pathogenesis of MS was subsequently confirmed in a study in which we demonstrated that the reduction of TG2 activity in a rat model for MS resulted in clinical improvement. The improvement coincided with reduced demyelination, reduced production of inflammatory mediators and less monocytes infiltrating into the CNS. These results thus support a role for TG2 in the adhesion, extravasation, and migration of monocytes during MS pathogenesis (van Strien et al. 2015).

Overall, our findings support a contributing role for TG2 to the pathogenesis of experimental models for MS resulting in monocyte infiltration into the CNS, which is a key factor in the development of clinical symptoms (Lassmann 2011). Future research should address the expression and function of TG2 in monocytes and macrophages derived from patients with ongoing MS.

## Considerations and conclusion

After tissue damage or infection, monocytes and monocyte-derived macrophages contribute significantly to the subsequent inflammatory reaction. To ensure a proper response, monocytes undergo several morphological and functional changes, which will allow them to adhere, extravasate, and migrate into the inflamed tissue. Subsequently, they differentiate into mature macrophages and, in addition to local tissue macrophages, kill the pathogens and/or dampen the inflammatory reaction, remove dead cells, and ultimately induce tissue repair. Over the last decades, it has been suggested that the multifunctional enzyme TG2 can participate, at least partially, in some of the above-mentioned processes under physiological conditions. Nowadays, an increased body of evidence reported that the expression and/or activity of TG2 can be regulated by inflammatory mediators, known to be released upon tissue damage or infection. Consequently, it should be considered that inflammation-driven TG2 can affect various responses of monocytes and macrophages under pathological conditions. In this review, we described the state of the art regarding the role of monocyte- and macrophage-derived TG2 in adhesion/extravasation, differentiation, and efferocytosis under inflammatory and pathological conditions with a keen eye on the inflammatory stimuli-regulating TG2 expression and function.

While reviewing the literature on this topic, the contribution of TG2 to inflammation-related processes performed by monocytes and macrophages becomes evident. However, it is often unclear, or unexplored yet, how TG2 affects these processes. Further research is needed to investigate the enzymatic (crosslinking) versus non-enzymatic activities of TG2, as well as the subcellular localizations involved. Most processes discussed in this review actually rely on the presence of cell surface or extracellular TG2 to sort its effect. It is known that inflammatory mediators can enhance surface TG2 on various cell types, including rat macrophages (Lisak et al. 2011; van Strien et al. 2011, 2015), thereby making TG2 easily accessible as a target for intervention. Another interesting aspect to consider is the observation that both pro- and anti-inflammatory mediators can regulate TG2 expression in monocytes and macrophages. So far, it is unknown if TG2’s regulation in expression coincides with the time-dependent processes occurring during inflammation, i.e. (1) pro-inflammatory mediators being present mostly in the early stage of inflammation and (2) anti-inflammatory mediators being present at the resolution stage of inflammation. Furthermore, it is not clear whether all monocytes and macrophages are stimulated to produce TG2 or just certain subpopulations. If the latter is true, this may facilitate inflammation-mediated responses in just a fraction of the cells. The defined gaps in knowledge are, at least partially, due to the fact that most studies described have been performed in vitro, lacking the complexity of an in vivo inflammatory response. Furthermore, contrasting data have been published on the role of TG2 in inflammation-related responses and in diseased conditions. Therefore, it is difficult to establish how and when TG2 is a beneficial or detrimental factor in inflammatory or in diseased conditions. To find adequate answers, more studies on relevant animal models for disease, as well as clinical studies on human subjects (or on human material) should be performed to include all the various stages of an in vivo inflammatory response and determine the role of TG2 in inflammation-related responses.

Based on the studies and considerations reported in this review, we conclude that monocyte- and macrophage-derived TG2 is clearly involved in various processes contributing to inflammation. However, TG2’s potential as a therapeutic target to counteract the possible detrimental effects or stimulate the potential beneficial effects in monocyte and macrophage responses during inflammation should be carefully considered. Alternatively, it is well known that TG2-related parameters can be used as a marker of disease, e.g., in celiac disease (Kneepkens and von Blomberg 2012) or of disease stage, e.g. in cancer (Eckert et al. 2015; Bravaccini et al. 2014). We thus put forward that this could be subject of research for monocyte- and macrophage-derived TG2 in inflammatory diseases including sepsis, atherosclerosis, and MS.

## Abbreviations

CNS: Central nervous system
ECM: Extracellular matrix
ERE: Estrogen responsive element
ER-α: Estrogen receptor alpha
FAK: Focal adhesion kinase
IFN-γ: Interferon gamma
IL-1β: Interleukin 1 beta
LPS: Lipopolysaccharide
M1: Classically activated/pro-inflammatory macrophages
M2: Alternatively activated, anti-inflammatory macrophages
MS: Multiple sclerosis
MTA1: Metastatic tumour antigen 1
PAF: Platelet-activating factor
ROCK: Rho-associated protein kinase
ROS: Reactive oxygen species
TG2: Tissue transglutaminase
TGF-β: Transforming growth factor beta
TNF-α: Tumour necrosis factor alpha
TLR4: Toll-like receptor 4

## References

[CR1] Achyuthan KE, Greenberg CS (1987). Identification of a guanosine triphosphate-binding site on guinea pig liver transglutaminase. Role of GTP and calcium ions in modulating activity. J Biol Chem.

[CR2] Aeschlimann D, Thomazy V (2000). Protein crosslinking in assembly and remodelling of extracellular matrices: the role of transglutaminases. Connect Tissue Res.

[CR3] Agnihotri N, Mehta K (2016). Transglutaminase-2: evolution from pedestrian protein to a promising therapeutic target. Amino Acids.

[CR4] Akimov SS, Belkin AM (2001). Cell-surface transglutaminase promotes fibronectin assembly via interaction with the gelatin-binding domain of fibronectin: a role in TGFbeta-dependent matrix deposition. J Cell Sci.

[CR5] Akimov SS, Belkin AM (2001). Cell surface tissue transglutaminase is involved in adhesion and migration of monocytic cells on fibronectin. Blood.

[CR6] Akimov SS, Krylov D, Fleischman LF, Belkin AM (2000). Tissue transglutaminase is an integrin-binding adhesion coreceptor for fibronectin. J Cell Biol.

[CR7] Balajthy Z, Csomos K, Vamosi G, Szanto A, Lanotte M, Fesus L (2006). Tissue-transglutaminase contributes to neutrophil granulocyte differentiation and functions. Blood.

[CR8] Balklava Z, Verderio E, Collighan R, Gross S, Adams J, Griffin M (2002). Analysis of tissue transglutaminase function in the migration of Swiss 3T3 fibroblasts: the active-state conformation of the enzyme does not affect cell motility but is important for its secretion. J Biol Chem.

[CR9] Boisvert WA, Rose DM, Boullier A, Quehenberger O, Sydlaske A, Johnson KA, Curtiss LK, Terkeltaub R (2006). Leukocyte transglutaminase 2 expression limits atherosclerotic lesion size. Arterioscler Thromb Vasc Biol.

[CR10] Bravaccini S, Tumedei MM, Scarpi E, Zoli W, Rengucci C, Serra L, Curcio A, Buggi F, Folli S, Rocca A, Maltoni R, Puccetti M, Amadori D, Silvestrini R (2014). New biomarkers to predict the evolution of in situ breast cancers. Biomed Res Int.

[CR11] DeFife KM, Jenney CR, Colton E, Anderson JM (1999). Cytoskeletal and adhesive structural polarizations accompany IL-13-induced human macrophage fusion. J Histochem Cytochem.

[CR12] Delhase M, Kim SY, Lee H, Naiki-Ito A, Chen Y, Ahn ER, Murata K, Kim SJ, Lautsch N, Kobayashi KS, Shirai T, Karin M, Nakanishi M (2012). TANK-binding kinase 1 (TBK1) controls cell survival through PAI-2/serpinB2 and transglutaminase 2. Proc Natl Acad Sci USA.

[CR13] Eckert RL, Fisher ML, Grun D, Adhikary G, Xu W, Kerr C (2015). Transglutaminase is a tumor cell and cancer stem cell survival factor. Mol Carcinog.

[CR14] Eligini S, Brioschi M, Fiorelli S, Tremoli E, Banfi C, Colli S (2015). Human monocyte-derived macrophages are heterogenous: proteomic profile of different phenotypes. J Proteomics.

[CR15] Eligini S, Fiorelli S, Tremoli E, Colli S (2016). Inhibition of transglutaminase 2 reduces efferocytosis in human macrophages: role of CD14 and SR-AI receptors. Nutr Metab Cardiovasc Dis.

[CR16] Espitia Pinzon N, Stroo E, t Hart BA, Bol JG, Drukarch B, Bauer J, van Dam AM (2014). Tissue transglutaminase in marmoset experimental multiple sclerosis: discrepancy between white and grey matter. PLoS One.

[CR17] Fadok VA, Bratton DL, Konowal A, Freed PW, Westcott JY, Henson PM (1998). Macrophages that have ingested apoptotic cells in vitro inhibit proinflammatory cytokine production through autocrine/paracrine mechanisms involving TGF-beta, PGE2, and PAF. J Clin Invest.

[CR18] Falasca L, Iadevaia V, Ciccosanti F, Melino G, Serafino A, Piacentini M (2005). Transglutaminase type II is a key element in the regulation of the anti-inflammatory response elicited by apoptotic cell engulfment. J Immunol.

[CR19] Falasca L, Farrace MG, Rinaldi A, Tuosto L, Melino G, Piacentini M (2008). Transglutaminase type II is involved in the pathogenesis of endotoxic shock. J Immunol.

[CR20] Fesus L, Piacentini M (2002). Transglutaminase 2: an enigmatic enzyme with diverse functions. Trends Biochem Sci.

[CR21] Fesus L, Sandor M, Horvath LI, Bagyinka C, Erdei A, Gergely J (1981). Immune-complex-induced transglutaminase activation: its role in the Fc-receptor-mediated transmembrane effect on peritoneal macrophages. Mol Immunol.

[CR22] Frisdal E, Lesnik P, Olivier M, Robillard P, Chapman MJ, Huby T, Guerin M, Le Goff W (2011). Interleukin-6 protects human macrophages from cellular cholesterol accumulation and attenuates the proinflammatory response. J Biol Chem.

[CR23] Gaudry CA, Verderio E, Jones RA, Smith C, Griffin M (1999). Tissue transglutaminase is an important player at the surface of human endothelial cells: evidence for its externalization and its colocalization with the beta(1) integrin. Exp Cell Res.

[CR24] Gentile V, Thomazy V, Piacentini M, Fesus L, Davies PJ (1992). Expression of tissue transglutaminase in Balb-C 3T3 fibroblasts: effects on cellular morphology and adhesion. J Cell Biol.

[CR25] Gerhardt T, Ley K (2015). Monocyte trafficking across the vessel wall. Cardiovasc Res.

[CR26] Ghanta KS, Pakala SB, Reddy SD, Li DQ, Nair SS, Kumar R (2011). MTA1 coregulation of transglutaminase 2 expression and function during inflammatory response. J Biol Chem.

[CR27] Giannone G, Sheetz MP (2006). Substrate rigidity and force define form through tyrosine phosphatase and kinase pathways. Trends Cell Biol.

[CR28] Gordon S (2003). Alternative activation of macrophages. Nat Rev Immunol.

[CR29] Gordon S, Taylor PR (2005). Monocyte and macrophage heterogeneity. Nat Rev Immunol.

[CR30] Gratchev A, Kzhyshkowska J, Utikal J, Goerdt S (2005). Interleukin-4 and dexamethasone counterregulate extracellular matrix remodelling and phagocytosis in type-2 macrophages. Scand J Immunol.

[CR31] Griffin M, Casadio R, Bergamini CM (2002). Transglutaminases: nature’s biological glues. Biochem J.

[CR32] Hasegawa G, Suwa M, Ichikawa Y, Ohtsuka T, Kumagai S, Kikuchi M, Sato Y, Saito Y (2003). A novel function of tissue-type transglutaminase: protein disulphide isomerase. Biochem J.

[CR33] Hayakawa K, Wang X, Lo EH (2016). CD200 increases alternatively activated macrophages through cAMP-response element binding protein—C/EBP-beta signaling. J Neurochem.

[CR34] Hodrea J, Demeny MA, Majai G, Sarang Z, Korponay-Szabo IR, Fesus L (2010). Transglutaminase 2 is expressed and active on the surface of human monocyte-derived dendritic cells and macrophages. Immunol Lett.

[CR35] Honing H, van den Berg TK, van der Pol SM, Dijkstra CD, van der Kammen RA, Collard JG, de Vries HE (2004). RhoA activation promotes transendothelial migration of monocytes via ROCK. J Leukoc Biol.

[CR36] Hsu TC, Chiang SY, Huang CY, Tsay GJ, Yang CW, Huang CN, Tzang BS (2007). Beneficial effects of treatment with transglutaminase inhibitor cystamine on macrophage response in NZB/W F1 mice. Exp Biol Med (Maywood, NJ).

[CR37] Huelsz-Prince G, Belkin AM, VanBavel E, Bakker EN (2013). Activation of extracellular transglutaminase 2 by mechanical force in the arterial wall. J Vasc Res.

[CR38] Iismaa SE, Mearns BM, Lorand L, Graham RM (2009). Transglutaminases and disease: lessons from genetically engineered mouse models and inherited disorders. Physiol Rev.

[CR39] Janiak A, Zemskov EA, Belkin AM (2006). Cell surface transglutaminase promotes RhoA activation via integrin clustering and suppression of the Src-p190RhoGAP signaling pathway. Mol Biol Cell.

[CR40] Johnson K, Hashimoto S, Lotz M, Pritzker K, Terkeltaub R (2001). Interleukin-1 induces pro-mineralizing activity of cartilage tissue transglutaminase and factor XIIIa. Am J Pathol.

[CR41] Jung SA, Lee HK, Yoon JS, Kim SJ, Kim CY, Song H, Hwang KC, Lee JB, Lee JH (2007). Upregulation of TGF-beta-induced tissue transglutaminase expression by PI3K-Akt pathway activation in human subconjunctival fibroblasts. Invest Ophthalmol Vis Sci.

[CR42] Kawabe K, Takano K, Moriyama M, Nakamura Y (2015). Lipopolysaccharide-stimulated transglutaminase 2 expression enhances endocytosis activity in the mouse microglial cell line BV-2. NeuroImmunoModulation.

[CR43] Khanna M, Chelladurai B, Gavini A, Li L, Shao M, Courtney D, Turchi JJ, Matei D, Meroueh S (2011). Targeting ovarian tumor cell adhesion mediated by tissue transglutaminase. Mol Cancer Ther.

[CR44] Kneepkens CM, von Blomberg BM (2012). Clinical practice: coeliac disease. Eur J Pediatr.

[CR45] Kuncio GS, Tsyganskaya M, Zhu J, Liu SL, Nagy L, Thomazy V, Davies PJ, Zern MA (1998). TNF-alpha modulates expression of the tissue transglutaminase gene in liver cells. Am J Physiol.

[CR46] Lassmann H (2011). Review: the architecture of inflammatory demyelinating lesions: implications for studies on pathogenesis. Neuropathol Appl Neurobiol.

[CR47] Laudanna C, Kim JY, Constantin G, Butcher E (2002). Rapid leukocyte integrin activation by chemokines. Immunol Rev.

[CR48] Lawrence T, Willoughby DA, Gilroy DW (2002). Anti-inflammatory lipid mediators and insights into the resolution of inflammation. Nat Rev Immunol.

[CR49] Lee J, Kim YS, Choi DH, Bang MS, Han TR, Joh TH, Kim SY (2004). Transglutaminase 2 induces nuclear factor-kappaB activation via a novel pathway in BV-2 microglia. J Biol Chem.

[CR50] Lesort M, Attanavanich K, Zhang J, Johnson GV (1998). Distinct nuclear localization and activity of tissue transglutaminase. J Biol Chem.

[CR51] Leu RW, Herriott MJ, Moore PE, Orr GR, Birckbichler PJ (1982). Enhanced transglutaminase activity associated with macrophage activation. Possible role in Fc-mediated phagocytosis. Exp Cell Res.

[CR52] Ley K, Laudanna C, Cybulsky MI, Nourshargh S (2007). Getting to the site of inflammation: the leukocyte adhesion cascade updated. Nat Rev Immunol.

[CR53] Lisak RP, Nedelkoska L, Studzinski D, Bealmear B, Xu W, Benjamins JA (2011). Cytokines regulate neuronal gene expression: differential effects of Th1, Th2 and monocyte/macrophage cytokines. J Neuroimmunol.

[CR54] Liu SF, Malik AB (2006). NF-kappa B activation as a pathological mechanism of septic shock and inflammation. Am J Physiol Lung Cell Mol Physiol.

[CR55] Lo CM, Wang HB, Dembo M, Wang YL (2000). Cell movement is guided by the rigidity of the substrate. Biophys J.

[CR56] Lorand L, Graham RM (2003). Transglutaminases: crosslinking enzymes with pleiotropic functions. Nat Rev Mol Cell Biol.

[CR57] Lorand L, Jeong JM, Radek JT, Wilson J (1993). Human plasma factor XIII: subunit interactions and activation of zymogen. Methods Enzymol.

[CR58] Lu S, Saydak M, Gentile V, Stein JP, Davies PJ (1995). Isolation and characterization of the human tissue transglutaminase gene promoter. J Biol Chem.

[CR59] Marchisio PC, Cirillo D, Teti A, Zambonin-Zallone A, Tarone G (1987). Rous sarcoma virus-transformed fibroblasts and cells of monocytic origin display a peculiar dot-like organization of cytoskeletal proteins involved in microfilament-membrane interactions. Exp Cell Res.

[CR60] Martinez FO, Helming L, Gordon S (2009). Alternative activation of macrophages: an immunologic functional perspective. Annu Rev Immunol.

[CR61] Martinez FO, Helming L, Milde R, Varin A, Melgert BN, Draijer C, Thomas B, Fabbri M, Crawshaw A, Ho LP, Ten Hacken NH, Cobos Jimenez V, Kootstra NA, Hamann J, Greaves DR, Locati M, Mantovani A, Gordon S (2013). Genetic programs expressed in resting and IL-4 alternatively activated mouse and human macrophages: similarities and differences. Blood.

[CR62] Matic I, Sacchi A, Rinaldi A, Melino G, Khosla C, Falasca L, Piacentini M (2010). Characterization of transglutaminase type II role in dendritic cell differentiation and function. J Leukoc Biol.

[CR63] Matlung HL, VanBavel E, van den Akker J, de Vries CJ, Bakker EN (2010). Role of transglutaminases in cuff-induced atherosclerotic lesion formation in femoral arteries of ApoE3 Leiden mice. Atherosclerosis.

[CR64] Mehta K, Lopez-Berestein G (1986). Expression of tissue transglutaminase in cultured monocytic leukemia (THP-1) cells during differentiation. Cancer Res.

[CR65] Mehta K, Lopez-Berestein G, Moore WT, Davies PJ (1985). Interferon-gamma requires serum retinoids to promote the expression of tissue transglutaminase in cultured human blood monocytes. J Immunol.

[CR66] Mehta K, Claringbold P, Lopez-Berestein G (1987). Suppression of macrophage cytostatic activation by serum retinoids: a possible role for transglutaminase. J Immunol.

[CR67] Metha K, Turpin J, Lopez-Berestein G (1987). Induction of tissue transglutaminase in human peripheral blood monocytes by intracellular delivery of retinoids. J Leukoc Biol.

[CR68] Mirza A, Liu SL, Frizell E, Zhu J, Maddukuri S, Martinez J, Davies P, Schwarting R, Norton P, Zern MA (1997). A role for tissue transglutaminase in hepatic injury and fibrogenesis, and its regulation by NF-kappaB. Am J Physiol.

[CR69] Mishra S, Murphy LJ (2004). Tissue transglutaminase has intrinsic kinase activity: identification of transglutaminase 2 as an insulin-like growth factor-binding protein-3 kinase. J Biol Chem.

[CR70] Miyamoto S, Akiyama SK, Yamada KM (1995). Synergistic roles for receptor occupancy and aggregation in integrin transmembrane function. Science.

[CR71] Moore KJ, Sheedy FJ, Fisher EA (2013). Macrophages in atherosclerosis: a dynamic balance. Nat Rev Immunol.

[CR72] Mosser DM, Edwards JP (2008). Exploring the full spectrum of macrophage activation. Nat Rev Immunol.

[CR73] Murphy KP, Travers P, Walport M, Janeway C (2008) Janeway’s immunobiology, vol v. 978, nos. 0-4129. Garland Science

[CR74] Murtaugh MP, Arend WP, Davies PJ (1984). Induction of tissue transglutaminase in human peripheral blood monocytes. J Exp Med.

[CR75] Muszbek L, Adany R, Mikkola H (1996). Novel aspects of blood coagulation factor XIII. I. Structure, distribution, activation, and function. Crit Rev Clin Lab Sci.

[CR76] Mycek MJ, Clarke DD, Neidle A, Waelsch H (1959). Amine incorporation into insulin as catalyzed by transglutaminase. Arch Biochem Biophys.

[CR77] Nadella V, Wang Z, Johnson TS, Griffin M, Devitt A (2015). Transglutaminase 2 interacts with syndecan-4 and CD44 at the surface of human macrophages to promote removal of apoptotic cells. Biochim Biophys Acta.

[CR78] Nakaoka H, Perez DM, Baek KJ, Das T, Husain A, Misono K, Im MJ, Graham RM (1994). Gh: a GTP-binding protein with transglutaminase activity and receptor signaling function. Science.

[CR79] Noseworthy JH, Lucchinetti C, Rodriguez M, Weinshenker BG (2000). Multiple sclerosis. N Engl J Med.

[CR80] Nurminskaya MV, Belkin AM (2012). Cellular functions of tissue transglutaminase. Int Rev Cell Mol Biol.

[CR81] O’Brien JM, Ali NA, Aberegg SK, Abraham E (2007). Sepsis. Am J Med.

[CR82] Pankov R, Yamada KM (2002). Fibronectin at a glance. J Cell Sci.

[CR83] Park KC, Chung KC, Kim YS, Lee J, Joh TH, Kim SY (2004). Transglutaminase 2 induces nitric oxide synthesis in BV-2 microglia. Biochem Biophys Res Commun.

[CR84] Park D, Choi SS, Ha KS (2010). Transglutaminase 2: a multi-functional protein in multiple subcellular compartments. Amino Acids.

[CR85] Rauhavirta T, Hietikko M, Salmi T, Lindfors K (2016). Transglutaminase 2 and transglutaminase 2 autoantibodies in celiac disease: a review. Clin Rev Allergy Immunol.

[CR86] Reinhart K, Bauer M, Riedemann NC, Hartog CS (2012). New approaches to sepsis: molecular diagnostics and biomarkers. Clin Microbiol Rev.

[CR87] Ribas V, Drew BG, Le JA, Soleymani T, Daraei P, Sitz D, Mohammad L, Henstridge DC, Febbraio MA, Hewitt SC, Korach KS, Bensinger SJ, Hevener AL (2011). Myeloid-specific estrogen receptor alpha deficiency impairs metabolic homeostasis and accelerates atherosclerotic lesion development. Proc Natl Acad Sci USA.

[CR88] Ritter SJ, Davies PJ (1998). Identification of a transforming growth factor-beta1/bone morphogenetic protein 4 (TGF-beta1/BMP4) response element within the mouse tissue transglutaminase gene promoter. J Biol Chem.

[CR89] Roberts CA, Dickinson AK, Taams LS (2015). The interplay between monocytes/macrophages and CD4(+) T cell subsets in rheumatoid arthritis. Front Immunol.

[CR90] Rose DM, Sydlaske AD, Agha-Babakhani A, Johnson K, Terkeltaub R (2006). Transglutaminase 2 limits murine peritoneal acute gout-like inflammation by regulating macrophage clearance of apoptotic neutrophils. Arthritis Rheum.

[CR91] Sarang Z, Koroskenyi K, Pallai A, Duro E, Melino G, Griffin M, Fesus L, Szondy Z (2011). Transglutaminase 2 null macrophages respond to lipopolysaccharide stimulation by elevated proinflammatory cytokine production due to an enhanced alphavbeta3 integrin-induced Src tyrosine kinase signaling. Immunol Lett.

[CR92] Sarkar NK, Clarke DD, Waelsch H (1957). An enzymically catalyzed incorporation of amines into proteins. Biochim Biophys Acta.

[CR93] Savill J (1997). Apoptosis in resolution of inflammation. J Leukoc Biol.

[CR94] Savill J, Dransfield I, Gregory C, Haslett C (2002). A blast from the past: clearance of apoptotic cells regulates immune responses. Nat Rev Immunol.

[CR95] Schnoor M (2015). Endothelial actin-binding proteins and actin dynamics in leukocyte transendothelial migration. J Immunol.

[CR96] Schuppan D, Hahn EG (2002). Biomedicine. Gluten and the gut-lessons for immune regulation. Science.

[CR97] Schwartz CJ, Valente AJ, Sprague EA, Kelley JL, Cayatte AJ, Mowery J (1992). Atherosclerosis. Potential targets for stabilization and regression. Circulation.

[CR98] Seiving B, Ohlsson K, Linder C, Stenberg P (1991). Transglutaminase differentiation during maturation of human blood monocytes to macrophages. Eur J Haematol.

[CR99] Shao WH, Cohen PL (2011). Disturbances of apoptotic cell clearance in systemic lupus erythematosus. Arthritis Res Ther.

[CR100] Sheetz MP, Felsenfeld DP, Galbraith CG (1998). Cell migration: regulation of force on extracellular-matrix–integrin complexes. Trends Cell Biol.

[CR101] Sospedra M, Martin R (2005). Immunology of multiple sclerosis. Annu Rev Immunol.

[CR102] Szondy Z, Sarang Z, Molnar P, Nemeth T, Piacentini M, Mastroberardino PG, Falasca L, Aeschlimann D, Kovacs J, Kiss I, Szegezdi E, Lakos G, Rajnavolgyi E, Birckbichler PJ, Melino G, Fesus L (2003). Transglutaminase 2^−/−^ mice reveal a phagocytosis-associated crosstalk between macrophages and apoptotic cells. Proc Natl Acad Sci USA.

[CR103] Thomas-Ecker S, Lindecke A, Hatzmann W, Kaltschmidt C, Zanker KS, Dittmar T (2007). Alteration in the gene expression pattern of primary monocytes after adhesion to endothelial cells. Proc Natl Acad Sci USA.

[CR104] Toth B, Garabuczi E, Sarang Z, Vereb G, Vamosi G, Aeschlimann D, Blasko B, Becsi B, Erdodi F, Lacy-Hulbert A, Zhang A, Falasca L, Birge RB, Balajthy Z, Melino G, Fesus L, Szondy Z (2009). Transglutaminase 2 is needed for the formation of an efficient phagocyte portal in macrophages engulfing apoptotic cells. J Immunol.

[CR105] Tovar-Vidales T, Clark AF, Wordinger RJ (2011). Transforming growth factor-beta2 utilizes the canonical Smad-signaling pathway to regulate tissue transglutaminase expression in human trabecular meshwork cells. Exp Eye Res.

[CR106] Turner PM, Lorand L (1989). Complexation of fibronectin with tissue transglutaminase. Biochemistry.

[CR107] Van Herck JL, Schrijvers DM, De Meyer GR, Martinet W, Van Hove CE, Bult H, Vrints CJ, Herman AG (2010). Transglutaminase 2 deficiency decreases plaque fibrosis and increases plaque inflammation in apolipoprotein-E-deficient mice. J Vasc Res.

[CR108] van Strien ME, Breve JJ, Fratantoni S, Schreurs MW, Bol JG, Jongenelen CA, Drukarch B, van Dam AM (2011). Astrocyte-derived tissue transglutaminase interacts with fibronectin: a role in astrocyte adhesion and migration?. PLoS One.

[CR109] van Strien ME, de Vries HE, Chrobok NL, Bol JG, Breve JJ, van der Pol SM, Kooij G, van Buul JD, Karpuj M, Steinman L, Wilhelmus MM, Sestito C, Drukarch B, Van Dam AM (2015). Tissue Transglutaminase contributes to experimental multiple sclerosis pathogenesis and clinical outcome by promoting macrophage migration. Brain Behav Immun.

[CR110] Wilhelmus MM, Verhaar R, Andringa G, Bol JG, Cras P, Shan L, Hoozemans JJ, Drukarch B (2011). Presence of tissue transglutaminase in granular endoplasmic reticulum is characteristic of melanized neurons in Parkinson’s disease brain. Brain Pathol.

[CR111] Wilhelmus MM, de Jager M, Bakker EN, Drukarch B (2014). Tissue transglutaminase in Alzheimer’s disease: involvement in pathogenesis and its potential as a therapeutic target. J Alzheimer’s Dis.

[CR112] Yakubov B, Chen L, Belkin AM, Zhang S, Chelladurai B, Zhang ZY, Matei D (2014). Small molecule inhibitors target the tissue transglutaminase and fibronectin interaction. PLoS One.

[CR113] Yamaguchi M, Zacharia J, Laidlaw TM, Balestrieri B (2016). PLA2G5 regulates transglutaminase activity of human IL-4-activated M2 macrophages through PGE2 generation. J Leukoc Biol.

[CR114] Yang J, Zhang L, Yu C, Yang XF, Wang H (2014). Monocyte and macrophage differentiation: circulation inflammatory monocyte as biomarker for inflammatory diseases. Biomark Res.

[CR115] Yen JH, Yang DJ, Chen MC, Hsieh YF, Sun YS, Tsay GJ (2010). Glycine tomentella Hayata inhibits IL-1beta and IL-6 production, inhibits MMP-9 activity, and enhances RAW264.7 macrophage clearance of apoptotic cells. J Biomed Sci.

[CR116] Yoo H, Ahn ER, Kim SJ, Lee SH, Oh SH, Kim SY (2013). Divergent results induced by different types of septic shock in transglutaminase 2 knockout mice. Amino Acids.

